# Modeling and predicting the growth of the mussel, *Mytilus edulis*: implications for planning of aquaculture and eutrophication mitigation

**DOI:** 10.1002/ece3.1823

**Published:** 2015-12-02

**Authors:** Per Bergström, Susanne Lindegarth, Mats Lindegarth

**Affiliations:** ^1^ Department of Marine Sciences – TjärnöUniversity of GothenburgTjärnö SE‐452 96StrömstadSweden

**Keywords:** Bivalve, ecosystem function, growth, mapping, marine spatial planning, *Mytilus edulis*, predictive modeling

## Abstract

The increased pressure on the marine ecosystems highlights the need for policies and integrated approaches for sustainable management of coastal areas. Spatial planning based on geographic information of human activities, ecological structures and functions, and their associated goods and services is a fundamental component in this context. Here, we evaluate the potential of predictive modeling to provide spatial data on one ecosystem function, mussel growth for use in such processes. We developed a methodology based on statistical modeling, spatial prediction, and mapping for the relative growth of the blue mussel, *Mytilus edulis*. We evaluated the performance of different modeling techniques and classification schemes using empirical measurements of growth from 144 sampling sites and data on biological, chemical, and physical predictors. Following comparisons of the different techniques and schemes, we developed random forest models to predict growth along the Swedish west coast. Implemented into GIS the best model produced in this study predicts that low, intermediate, and high growth rates can be expected in 53%, 32%, and 15% of modeled area, respectively. The results of this study also suggest that the nature and quality of predictor data hold the key to improving the predictive power of models. On a more general note, this study exemplifies a feasible approach based on measuring, modeling, and mapping for obtaining scientifically based spatial information on ecosystem functions and services affected by a complex set of factors. Such information is fundamental for maritime spatial planning and ecosystem‐based management and its importance is likely to increase in the future. Because of its close link to nutrient assimilation and production yield, site‐specific information of soft tissue growth such as the map of predicted growth rate developed in this study can be used as a tool for optimizing actions aimed at mitigating eutrophication and aquaculture operations and in maritime spatial planning processes of coastal areas.

## Introduction

Increasing human pressure on the marine environment highlights the need for management and planning of the marine resources (Margules and Pressey 2000). This is reflected in numerous legal and policy documents that are developed around the world (e.g., the EU “Roadmap for marine spatial planning,” COM 2008; and the recently adopted “Framework directive of maritime spatial planning,” European Commission 2014). Combining ecosystem‐based management with marine spatial planning and ecosystem service framework is a valuable way to ensure stability of marine systems and their services (Guerry et al. 2012). This approach relies heavily upon access to reliable spatial information of structural and functional biodiversity and the knowledge of human pressures.

To improve management and planning there is a need for information on system functions and processes (Troy and Wilson 2006; Frid et al. 2008). The understanding of how these processes work and how they respond to changes are continuously increasing (Sutherland et al. 2013). However, such data are primarily collected using small‐scale sampling methods resulting in maps that are constructed using direct methods often being incomplete or nonexistent. As a consequence of this, and the fact that few species have been studied in detail in terms of their dynamic response to environmental changes, decisions about planning and resource‐use are often based on incomplete information (Toner and Keddy 1997; Joy and Death 2004).

Methods utilizing the relationship between a biological response (e.g., growth, abundance) and explanatory environmental variables have been increasingly used to fill gaps in information. Predictive models are powerful tools in combining field surveys with these kinds of relationships. This has led to an increased use of models to predict distribution and abundance of species in natural sciences across terrestrial and aquatic environments (Guisan and Zimmermann 2000; Elith and Leathwick 2009). The same principle of predictive mapping that have extensively been used in terrestrial environments also applies in underwater environments (Remillard and Welch 1993). The gaps in information have led to models and maps, based on the models, being sought after by scientists, conservation, and management planners as valuable tools in the management of terrestrial and aquatic ecosystems (Toner and Keddy 1997; Schmolke et al. 2010).

Recent developments in geographical information system (GIS) and data storage have facilitated the use of predictive models across space for use in spatial analysis and for the production of maps. The ability of GIS to integrate spatial data and visualize results has proved essential for landscape‐scale analyses (Frohn 1998; Johnston 1998) and become an important tool for managers investigating environments on a landscape‐scale (Remillard and Welch 1993; Ferguson and Korfmacher 1997). Although historically mainly used in terrestrial ecology (Johnston 1998), GIS is now used in wide range of applications including the marine environment (Scott et al. 2002). Combining predictive modeling with the production of maps can provide invaluable information for conservation, spatial planning, and management of marine coastal areas.

Although aquaculture is not a new concept, the increased human activities in coastal environments in combination with growing importance of aquaculture in food production (Bostock et al. 2010) have resulted in increased demands for planning and sustainability of aquaculture activities. Since filter feeders are near the base of the food web and only rely on naturally available food (Crawford et al. 2003), cultivation of filter‐feeding organisms is probably one of the more environmental friendly marine production system available (Shumway et al. 2003). This has led to proposals that mussel farming could be used as a tool in mitigation of eutrophic coastal areas and at the same time offer sustainable seafood production (Lindahl et al. 2005). Such tools may be of particular importance as components in programs of measures for achieving “good environmental status” as requested by the EU Water Framework Directive (WFD).

Growth of bivalves and thus their potential as mitigators of eutrophication effects depends on a complex matrix of biological, physical, and geomorphological properties (Dame 1996). In an applied context, efficient management of mussel farming requires geographically explicit knowledge about differences in growth among areas. Comprehensive studies of bivalve growth, like studies on almost everything in the oceans, are practically impossible. A wide range of models, from empirical (Gangnery et al. 2003; Hawkins et al. 2004) to mechanistic (Saraiva et al. 2011; Thomas et al. 2011) has been used to model growth of bivalves. Predictive models and maps showing the spatial distribution of mussel growth serve many purposes, including developing aquaculture production, supporting spatial planning, management and designing experiment to test mussel farming as a mitigation tool, and has successfully been applied to evaluate potential new mariculture sites (e.g., Brigolin et al. 2006; Filgueira et al. 2010) and manage impacts (e.g., Grangeré et al. 2010).

The overall aims of this study were to evaluate and test the predictability of spatial growth patterns of the common bivalve *Mytilus edulis* Linnaeus 1758, on the Swedish west coast. This information is of fundamental importance for the ongoing development and planning of aquaculture activities, both for human consumption and as a measure to mitigate eutrophication. A previous study by Bergström et al. (2013) indicates that spatial patterns of soft tissue growth are temporally consistent at certain spatial scales, while those of shell growth were not. This implies that there are static factors, which lead to spatial patterns soft tissue growth that are potentially predictable, provided that these factors or proxies thereof can be identified. Thus, we hypothesized that spatial patterns of relative soft tissue growth will be to some extent predictable and possible to map in a meaningful way using geographical and environmental predictor variables. Spatial patterns of shell growth, however, will not be predictable in the same way. These aims are achieved by (1) measurement of growth of approximately 2000 transplanted mussels at >100 representative locations along the Swedish west coast during four periods within 3 years, (2) evaluation of model performance using a range of different modeling methods, classification schemes, and predictor variables, and (3) spatial prediction and mapping of growth using selected methods and models.

## Materials and Methods

### Study area and growth experiments

**Figure 1:**
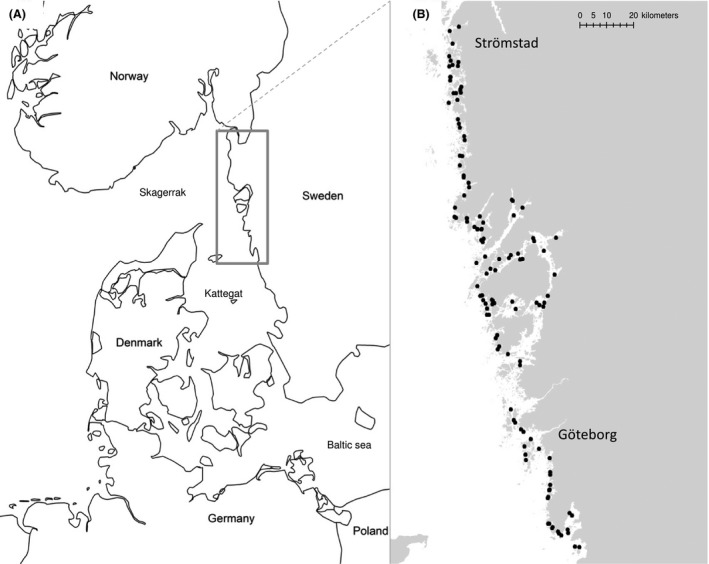
Geographic location of sampling area (A) with specification (B) of the randomly selected sites where growth was estimated using transplanted mussels.

The modeling in this study is based on growth data of *Mytilus edulis* collected during three successive years, 2010–2012, in the waters from Strömstad (58°56′N, 11°10′E) to the Kungsbacka fjord (57°22′N, 12°03′E). Growth of *M. edulis* was measured, using transplanted mussels, over 2 months during four separate experimental periods (2010: September–November; 2011: May–July and August–October, and 2012: June–August). Following the sampling strategy design developed by Bergström et al. (2013), 25 mussels were kept in semisoft plastic cage (25 × 10 × 10 cm, mesh size 10 mm) tied to concrete blocks and buoyed to float submerged at 2 m below surface at each site. From each cage, 15 mussels were randomly selected for growth measurements. Data on dry soft tissue (DST) and shell length (SL) were collected and the growth of individual mussels estimated as the difference in DST and SL between the individual measurements of transplanted mussels and the average of a starting pool consisting of 100 mussels. Using this approach the expected precision of the measurements among mussels and sites were on average using standard error estimation about 10% of the expected growth (Bergström et al. 2013). In total, growth was measured for 144 sites within the water depth of 6–20 m (Fig. 1). The sampling sites were randomly selected within areas corresponding to water bodies defined by Swedish authorities in compliance with the WFD and the sampling covered almost every water body within the investigated area. To eliminate differences in growth due to age or size, all transplanted mussels originated from the same location and were of the same start size range (40–50 mm) and age.

### Spatial and temporal variability in growth

Establishing significant spatial patterns of growth is a prerequisite for predictive or mechanistic modeling. Furthermore, it is highly likely that there are systematic differences in growth among experimental periods. Therefore, having sampled different sites within each period, initial analyses of spatial and temporal patterns of growth were analyzed using a nested ANOVA (Underwood 1997). Following the results of these analyses, which indicated substantial differences in average growth among experimental periods, data were standardized among periods using a *z*‐transformation (i.e., $Z_{ij}=\left(x_{ij}-{\overset{¯}{x}}_{i}\right)/s_{i}$), where *x*_*i*j_ is the *j*th measurement in the *i*th period. This transformation successfully removed variability among periods, while maintaining differences among sites.

### Predictive modeling of growth

#### Classification of growth

**Figure 2:**
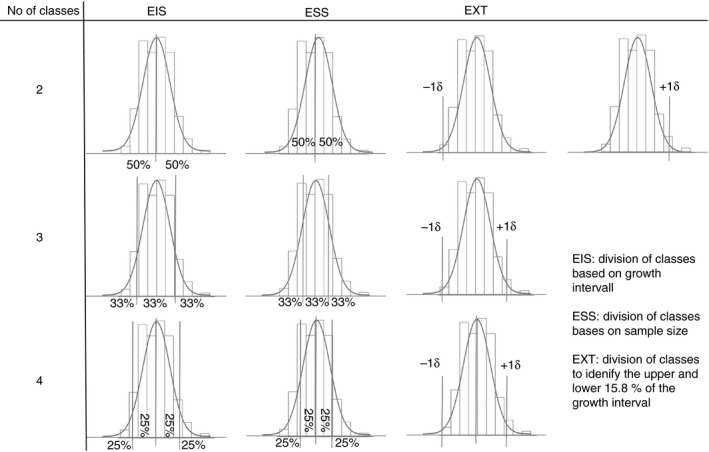
Classification schemes used for the four modeling techniques used.

As explained above, prediction of growth in absolute terms was made difficult by substantial differences in growth among periods. Nevertheless, prediction of spatial patterns of relative growth was potentially possible. Therefore, the growth data were classified with a varying degree of detail and according to three different rules. The number of classes varied from two to four, and each of these were classified according to three different schemes: equal size intervals (EIS), equal sample sizes per class (ESS), and identification of sites with extremely high and low growth (EXT) (Fig. 2).

#### Selection of predictors

In order to model, predict, and ultimately map spatial patterns of growth classes, full covering data on predictor variables are needed. We attempted different variables as predictors. While geographical and oceanographical parameters could be derived from national databases (Naturvårdsverket 2006; SMHI 2014) and digital nautical charts in GIS, coupling hydrological, chemical, and biological variables to experimental sites was more difficult. Considering the large number of stations and the expected large variability in small temporal scales direct measurement at periods of sampling was not deemed plausible. Instead we used two sets of analyses a priori to evaluate the usefulness of modeled data from the area. In the first step, we used data from 12 years and 13 water bodies to analyze the relationship between measured data and modeled environmental data from “The Coastal Zone Model” (Sahlberg 2009) which is part of the model system used by Swedish Meteorological and Hydrological Institute (SMHI). Second, we evaluated the temporal consistency of spatial patterns for these variables using measured data from 21 monitoring sites during 12 years. The rationale of these latter analyses was to identify variables showing consistent patterns among years suitable for predicting consistent spatial patterns of growth. Detailed accounts of these analyses are given in the supporting information. These procedures and removal of a number of strongly correlated predictors resulted in a list of 11 variables which were used for modeling (Table 1).

**Table 1:** Explanatory variables used in models after evaluating temporal consistency of spatial pattern, correlation between measured and modeled data, and variance inflation factor analysis (see supporting information for further details)

Category	Variable	Unit	Mean	SD	Min	Max
Geographical	Latitude^2^	°N	58.18	0.47	57.32	59.00
	Distance to baseline^2^	m	7785	7326	0	2.8*10^9^
Oceanographical	Area volume^1^	km^3^	0.13	0.26	0.001	1.8
	Turnover time^1^	days	17.5	35.1	0.24	184
	Exposure^3^	m^2^/s	45238	78713	407	536380
Hydrological	Temperature^1^	°C	13.6	2.87	8.80	17.9
	Salinity^1^	PSU	26.8	1.71	23.1	30.7
Biological	Chlorophyll *a* 1	mg/m^3^	3.05	0.59	1.99	5.25
Chemical	Ammonium^1^	mg/m^3^	0.83	0.97	0.11	7.63
	Total nitrogen^1^	mg/m^3^	224	25.1	192	313
	Total phosphorus^1^	mg/m^3^	17.2	3.21	13.5	37.0

Data sources: ^1^SMHI, ^2^Map, and ^3^SAKU.

#### Modeling techniques

To model the growth of *M. edulis*, we used four techniques which are commonly used in species and habitat distribution modeling to model relationships between growth and environmental factors: random forest (RF; Breiman 2001a; Liaw and Wiener 2012), generalized additive models (GAM; Hastie and Tibshirani 1986), multivariate adaptive regression splines (MARS; Friedman 1991), and conditional inference forest (CI; Hothorn et al. 2006).

The modeling was done in two steps. First, predictability of growth classes for DST and SL was compared among techniques. This was to test the hypothesis that spatial patterns of DST are more predictable than those of SL and to select the most efficient method for further modeling. This was done by randomly splitting the original dataset of 144 data points into a training dataset containing 70% of the data from each class for fitting the models. The test dataset (remaining 30% of samples) was used to give an independent estimate (hereafter referred to as external evaluation) of the predictive power of models (Verbyla and Litvaitis 1989), whereas the training dataset was used for internal validation of the models. This procedure was repeated 1000 times to achieve a stable and confident estimate of the model performance. Second, based on the conclusions about differences among methods and response variables from the previous tests among methods (see “Predictive modeling of growth” section), models were fitted using the whole dataset to evaluate differences among classification schemes and variable importance. This was justified by the small differences between internal and external performance criteria and by the fact that a larger number of data are likely to produce better models (nevertheless the initial external validation was necessary to assess and statistically test their performance).

The performance of models using different classification schemes and methods in the steps above was measured as the area under curve (AUC) and other accuracy criteria (i.e., accuracy, accuracy vs. no information ratio (ACC/NIR), sensitivity, and specificity; Fielding and Bell 1997). The AUC measures how well a parameter can distinguish between two diagnostic groups while sensitivity is used to evaluate the proportion of a class that is correctly classified and specificity measures the number of correctly classified absences out of all absences. Accuracy is the number of correctly classified presences and absences and comparing the observed accuracy to a random assignment of classes generates ACC/NIR. Variable importance was also used to evaluate the important processes and how the different modeling (i.e., different classification schemes) influenced the importance of explanatory variables.

All analyses were done using purpose‐built scripts using the R software (R Core Team 2014), complemented by the following packages: “lme4” (Bates et al. 2014) for lmer and “randomForest” (Liaw and Wiener 2012), “mgcv” (Wood 2013), “earth” (Milborrow 2014), and “party” (Hothorn et al. 2013) for random forest, GAM, MARS, and CI analysis, respectively, and 1000 trees were built to calculate the average tree to obtain stable results. Predictions of mussel growth in unmeasured areas were generated in R using the package “stats” and ArcGIS 10.1 (ESRI 2012) was thereafter used to create maps using the predictions.

## Results

### Spatial and temporal variability in growth

**Figure 3:**
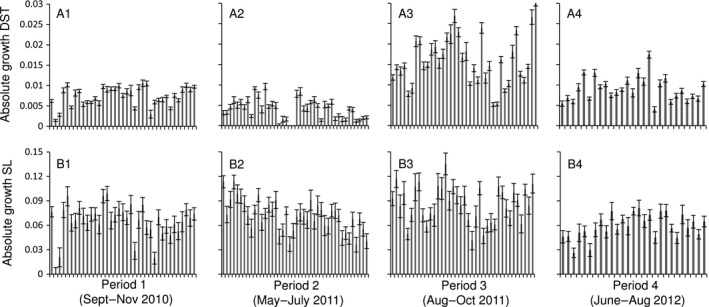
Comparison of absolute growth of (A) DST and (B) SL for four sampling periods (1–4) with sampling locations arranged in north–south direction from left to right within each period.

Quantitative analyses showed that there was significant variability in growth among both sites and periods for shell length and dry soft tissue (Table 2, absolute growth). Inspection of means show that the largest growth rates were observed in the third period for both DST and SL, with maximum growth rates at 0.03 g and 0.14 mm per day (Fig. 3). The smallest growth rates for DST were observed in the second period while growth in SL was lowest in the first and fourth period. Analyses of variance components showed that there were large differences in the relative importance in sources of variability between DST and SL. For DST, the variability among periods and sites were both substantially larger than among individual mussels, while for SL the variability among individual mussels was 5–10 times larger than among periods and sites (Table 2). Thus, despite the observation of significant variability among periods and sites for both measures of growth, it is clear that spatial and temporal patterns of DST are substantially stronger than those of SL. Nevertheless, in order to assess models and predict spatial patterns of growth, both DST and SL were standardized using the procedures described in “Spatial and temporal variability in growth” section. As expected, this standardization successfully removed variability among periods, while maintaining the spatial structure (Table 2, relative growth).

**Table 2:** Analysis of variance of mean growth in soft tissue and shell length using absolute and relative (*z* standardized) measurements of growth

Source		Absolute growth				Relative growth		
	df	MS (10^−5^)	F	*P*	VC (10^−5^)	MS	F	*P*
Soft tissue = DST								
Period	3	1497	69.2	<0.001	2.7	0	0	1
Site (period)	140	21.6	23.2	<0.001	1.4	8.77	19.2	<0.001
Residual	2005	0.93			0.93	0.46		
Length = SL								
Period	3	6607	11.6	<0.001	11.2	0	0	1
Site (period)	140	570	4.3	<0.001	29.1	3.38	4.05	<0.001
Residual	2005	113			133	0.83		

VC, variance component.

### Predictive modeling of growth

#### Assessment of predictability of growth and selection of modeling technique

The initial set of models, used to compare the predictability of growth of DST and SL using a range of techniques and classification schemes, showed clear and consistent differences among variables and methods (Table 3). First, for both AUC and ACC/NIR the performance of models of DST (based on external validation) was generally better than those of SL. The best models of DST produced AUC values of 0.73 and an accuracy of up to 65% (i.e., ACC/NIR = 1.65) better than at random (Table 3). The corresponding performance was 0.66% and 29% for the best models of SL.

**Table 3:** Model performance for soft tissue and length using external validation and the measurements of AUC, accuracy versus no information ratio (ACC/NIR), and the mean *P‐*value for 1000 runs of each model and classification combination. EIS, equal interval size; ESS, equal sample size; EXT, extremes. Significant models in bold

Technique	No of classes	Scheme	AUC		ACC/NIR	
			DST	SL	DST	SL
RF	2	EIS	0.66	0.60	1.06	1.01
		ESS	0.73	0.56	1.46	1.10
		EXT	0.59	0.51	1.00	0.99
	3	EIS	0.66	0.66	1.19	0.97
		ESS	0.71	0.64	**1.65**	1.27
		EXT	0.65	0.52	1.01	0.99
	4	EIS	0.69	0.61	1.00	0.95
		ESS	0.68	0.62	**1.67**	1.29
		EXT	0.73	0.55	1.04	0.99
MARS	2	EIS	0.60	0.57	0.96	0.94
		ESS	0.62	0.58	1.23	1.15
		EXT	0.54	0.52	0.98	0.99
	3	EIS	0.63	0.62	1.09	0.92
		ESS	0.64	0.61	1.43	1.19
		EXT	0.60	0.55	0.97	0.97
	4	EIS	0.65	0.58	0.85	0.89
		ESS	0.63	0.61	1.22	1.22
		EXT	0.69	0.60	0.90	0.92
CI	2	EIS	0.50	0.50	1.00	1.00
		ESS	0.63	0.57	1.26	1.13
		EXT	0.50	0.50	1.00	1.00
	3	EIS	0.57	0.54	1.07	1.02
		ESS	0.66	0.60	1.39	1.21
		EXT	0.50	0.50	1.00	1.00
	4	EIS	0.52	0.50	0.98	0.99
		ESS	0.63	0.61	1.33	1.24
		EXT	0.50	0.50	0.98	1.00
GAM	2	EIS	0.49	0.49	0.95	0.98
		ESS	0.58	0.62	1.15	1.23
		EXT	0.50	0.50	0.98	1.00
	3	EIS	0.53	0.61	1.01	1.02
		ESS	0.51	0.60	1.08	1.05
		EXT	0.50	0.50	0.95	1.02
	4	EIS	0.52	0.51	0.79	0.36
		ESS	0.58	0.62	1.10	1.17
		EXT	0.49	0.52	0.92	0.30

**Figure 4:**
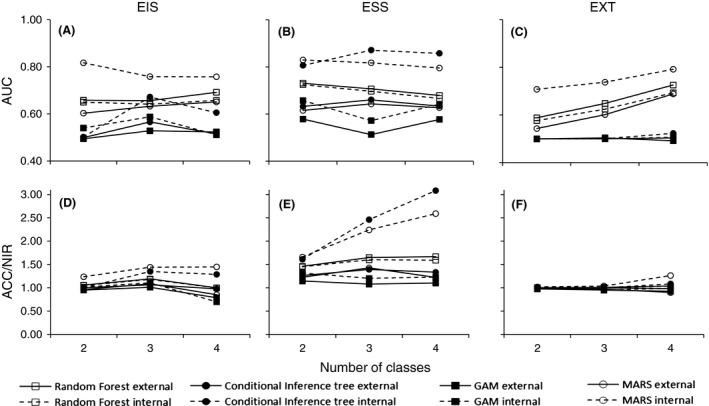
Summary of two model performance measurments AUC (A–C) and ACC/NIR (D–F) of growth in soft tissue using internal and external validation for four different modeling techniques (random forest, conditional inference, GAM, and MARS), three different number of classes (two, three, and four classes) and three classification schemes (EIS, ESS, and EXT).

Comparisons of the different modeling methods, measured as performance in internal validation, revealed that conditional inference trees (CI) and MARS produced the best fit to data (Fig. 4). These methods apparently produced AUC values >0.8 (Fig. 4) and accuracies more than twice as precise at random (Fig. 4). However, focusing on the performance in external validation a different pattern arose. Instead random forest (RF) models performed better than all other methods for DST growth irrespectively of performance measurement or classification used. No such pattern existed for growth in SL (Fig. 4, Table 3). The modeling techniques performed equally poorly for growth in SL with AUC values rarely exceeding 0.65 and only one model had a *P*‐value smaller than 0.2. In fact the only significant (*P* < 0.05) models using external validation were the RF models for DST growth (ESS classification for classes 3 and 4).

As expected, the AUC for different methods were consistently higher using internal validation than external. One important observation was the fact that for RF models the external AUC was of similar size (or higher) as for internal validation (Fig. 4). The RF models also showed the highest external AUC value (DST ~ 0.7; SL ~ 0.6), but still much variation in growth could not be explained using the available explanatory variables. The accuracy of models decreased with increasing complexity of the models. However, comparing the observed accuracy to a random assignment of classes (i.e., accuracy vs. no information ratio, ACC/NIR) it was clear that this mainly is an effect of different sizes of the classes (i.e., different number of sites among the classes). Compared to the decrease in accuracy, ACC/NIR tended to increase with increased complexity of the DST model (Fig. 4, Table 3) while remaining about the same for all models predicting SL (Table 3). Models of growth in SL performed were usually <15% better than at random. The best model performance was two RF models (3 classes ESS; 4 classes ESS), which were 27–29% better than random while for almost half (42%) of the cases the models did not add any predictive performance at all (ACC/NIR ≤1.00, Table 3). For DST growth prediction the models performed much better with between 50% and 100% (ESS models) and 30–50% (EIS) better than at random while for EXT only the four‐class model performed better (+38%) than random. This suggests that models based on classifications using equal sample sizes are most useful for the purpose of modeling mussel growth.

Using an overall assessment of the different methods and growth measurements, we concluded that further evaluation of growth models should be performed on DST using RF models and that the whole dataset could be used in creating the models due to the similarity in performance measurements between internal and external validation in this initial step.

#### Evaluation of classification schemes and variable importance for models of DST

**Figure 5:**
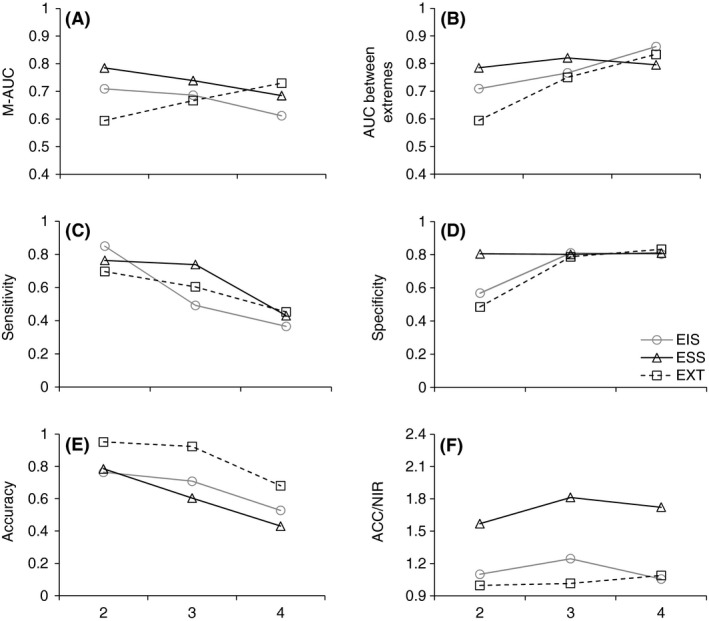
Performance of random forest models on dry soft tissue using data from all sites. (A) Multiple AUC (M‐AUC), (B) AUC between groups of extreme growth, (C) sensitivity, (D) specificity, (E) accuracy, and (F) ACC/NIR.

The subsequent analyses of RF models, using the whole dataset, showed that the EIS and ESS models follow the same general pattern while the EXT model differs slightly for some of the performance measurements (Fig. 5). Multiclass AUC (M‐AUC) is relatively stable, decreasing slightly with increasing number of classes while the ability to separate the classes with the highest and lowest growth increases slightly. Increased model complexity slightly increased the specificity for EIS and EXT models, while ESS model showed a stable specificity over different number of classes. The sensitivity decreased from roughly 0.75–0.4 when increasing the number of classes from two to four for all classification schemes (Fig. 5). The accuracy of models were highest (0.7–0.95) for EXT, but again adjusting for no information ratio (i.e., ACC/NIR) reduced the usefulness of these models as only the four‐class model was better (+9%) than random with EIS slightly better (5–20%), while ESS model was 50–80% better than random for all classes.

Weighing the different performance measures against each other clearly showed that for evaluation of variable importance an ESS or EIS model was most suitable and that three classes provided good compromise between the two‐class models that generally showed higher AUC and sensitivity and four‐class models that had higher specificity and ACC/NIR. Consequently, we evaluated the importance of explanatory variables on growth of DST for the random forest three‐class ESS model (1000 trees, 1000 runs, AUC = 0.71). Analysis of variable importance (mean decrease accuracy) showed that four variables, total phosphorous, salinity, latitude, and turnover, were the most important for the accuracy of the predictive models. Furthermore, visual inspection of partial dependencies showed that areas of high growth were associated with high turnover rates, high salinity, and high total phosphorous levels, while areas of low growth were associated with low salinity and low turnover. However, in terms of creating homogenous groups in the end nodes of the predictive models (i.e., finding and classifying sites with similar growth into the same predicted growth class) other variables such as exposure and distance to baseline were more important. These results suggest that different environmental parameters affect the growth of mussels in different ways. For example, salinity affects growth rates directly, while others (e.g., exposure) affect the growth indirectly by modifying other growth‐related parameters.

#### Mapping of relative growth of DST

**Figure 6:**
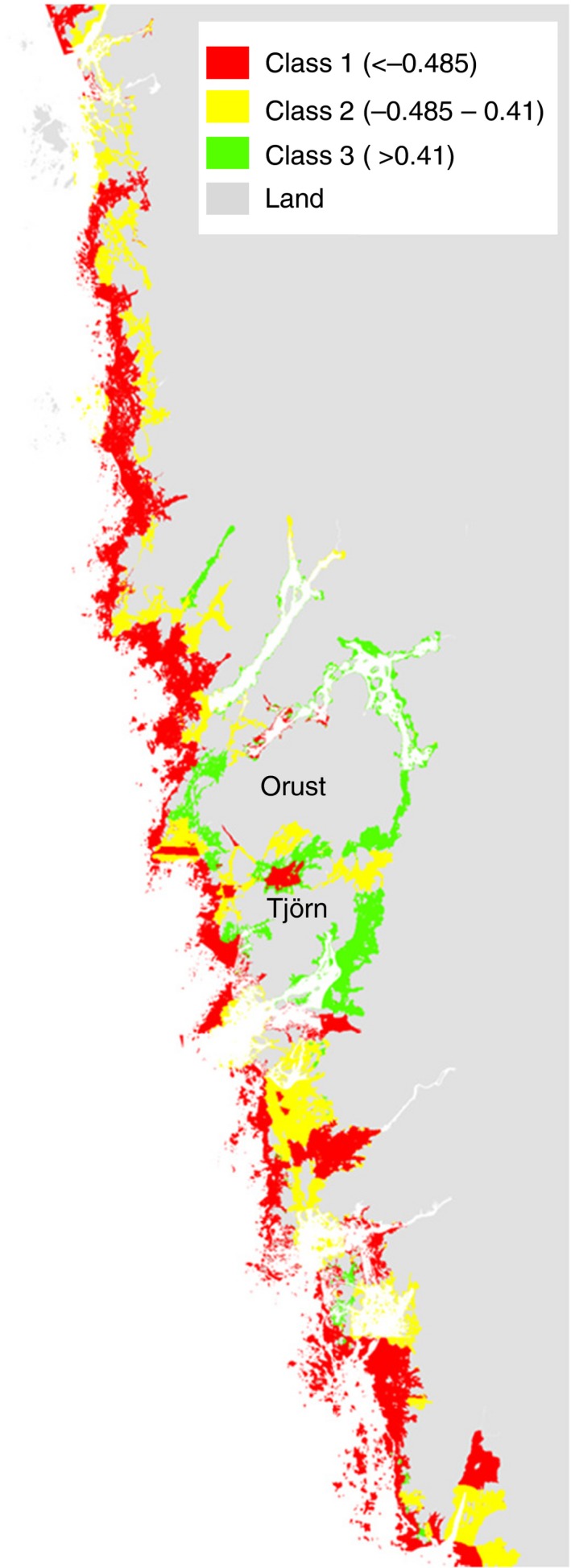
Predicted growth class using the three‐class ESS model for relative growth. Classes 1–3 represent lowest (red, <−0.485) to highest (green, >0.41) predicted relative growth while white areas are waters deeper than the investigated depth span (−20 m). Predicted class occurrence: class 1: 52.9%, class 2: 31.9%, and class 3: 15.2%.

Prediction and mapping of growth using the three‐class ESS model revealed a pattern with generally higher growth closer to shoreline and inside bays (Fig. 6). However, growth was reduced close to estuaries with outflows of large rivers. The areas with highest growth rates were found inside the fjord system around the large islands Orust and Tjörn in the center of the investigated area (Fig. 6). In total, slightly more than half of the area (52.9%) was predicted as belonging to the class with lowest growth rate (class 1), 31.9% as medium growth rate areas (class 2), and 15.2% of the area belonging to class with highest rate of mussel growth (i.e., class 3).

## Discussion

This study demonstrates a comprehensive strategy mapping of an ecological process, soft tissue growth of the blue mussel *M. edulis*, which is fundamental both to the economic benefits of mussel farming and to its efficiency as a method to mitigate eutrophication. Using an approach based on empirical measurements of growth on transplanted mussels and statistical modeling, we analyzed spatial patterns and tested the possibilities for predictive modeling and mapping of growth of the blue mussel *M. edulis*. We observed strong and significant spatial and temporal variability in growth of dry soft tissue and shell length, but as expected from earlier studies (i.e., Bergström et al. 2013) spatial patterns of growth of mussel tissue were more predictable than that of shell length. By removing temporal variability using standardization, we were able to classify and predict areas with relatively low, medium, and high growth rates of mussel tissue with an average accuracy, which was roughly twice as large as that of a random assignment of growth classes.

Although transplant experiments of biota and artificial substrates have been used to investigate a range of ecological patterns and processes (e.g., Ellis et al. 2002; Honkoop et al. 2003; Crain et al. 2004), and empirical models are becoming increasingly popular for spatial modeling and mapping of species, habitats (Reiss et al. 2011, 2014; Bučas et al. 2013), and even proxies of ecosystem services (e.g., Martínez‐Harms and Balvanera 2012; Brown and Fagerholm 2015); this is to our knowledge the first study to combine these two approaches in a comprehensive way. The results provide information about specific spatial and temporal patterns of growth as well as factors important for predicting the growth of mussels in this particular part of the Swedish coast. In a more general context, the study has the potential to provide more general insights into the approaches for spatial modeling and mapping of ecological functions and processes in the marine environment. As ecological processes link biodiversity to societal values, development of such approaches is of growing relevance as tools for marine spatial planning and for assessment of ecosystem services (e.g., Crossman et al. 2013; Hauck et al. 2013).

From the perspective of mussel farming on the Swedish west coast, the methodological approach used here provides robust scientifically based predictions of growth which can be used directly in planning of aquaculture operations in this particular coastal area. The formal methodology of the different steps of the process allowed objective testing of the performance of models, estimation of uncertainty, and can be used to gauge alternative approaches. As an example, estimates of the uncertainty of current models suggest that the models successfully separate classes of low, medium, and high growth with an accuracy that is considered good by scientific standards (e.g., AUC ≈ 0.75 on average and 0.8 among extremes). Furthermore, it is interesting to note that 47% of the available permissions for farming of *M. edulis* in Sweden are situated in areas predicted to be most favorable with respect to growth despite the fact that these areas only represent 15.2% of the total area. This may be an indication that the predicted spatial pattern of growth is consistent with experiential knowledge possessed by farmers and other stakeholders.

Notwithstanding the relative success of modeling, it is clear that there is also a substantial scope for improving the accuracy and resolution of models and maps. A wide array of flexible methods for statistical modeling of nonlinear and interactive relationships are available (e.g., Elith et al. 2006; Araújo and New 2007) and the analyses presented here suggest that the predictive power may differ among methods. Nevertheless, we suggest that aspects to do with predictor data, rather than the choice of modeling technique, are likely to be more fruitful for improving the predictive power in modeling efforts like these. In particular, we suggest that two aspects, (1) a stronger mechanistic coupling and (2) the matching between the spatial resolution of predictor and response are of general importance for the success of future modeling efforts.

First, because the aim of this study is primarily to provide validated and robust predictions of spatial and geographic patterns of mussel growth, to be used in a planning and management context, mechanistic understanding of causal links is not essential (e.g., Peters 1991; Breiman 2001b). In fact, correlative approach was deliberately selected in favor of analytical models because of the multitude of physical, chemical, and biological factors that might influence growth directly, indirectly, or interactively and because coherent data covering the whole area of these variables are not likely to be available. Nevertheless, it is clear that also the predictive power of models will benefit greatly by including predictors with close mechanistic direct or indirect links to growth (e.g., Elith and Leathwick 2009; Lindegarth et al. 2014).

Second, the predictive power of spatially explicit models can also be greatly influenced by the matching of resolution between predictor and response data and the scale of variability in important ecological processes (e.g., Tobalske 2002; Svensson et al. 2013). In this study, data on mussel growth was collected at individual sites in small‐scale (<1 m) units. These were matched with predictor data from 25 × 25 m GIS grids (e.g., wave‐exposure) and modeled data on hydrological, oceanographical, and chemical variables estimated at the scale of water bodies with a resolution of several kilometers (e.g., salinity and turnover). The rationale behind this strategy was that earlier analyses had shown that variability in growth of soft tissue was orders of magnitude larger among water bodies than within, and that spatial patterns were largely consistent among years at the scale of water bodies but not at the scale of sites (Bergström et al. 2013). This approach was further justified by initial analyses showing significant and occasionally strong correlations between monitoring and modeled data and analyses indicating temporal consistency of spatial patterns for many of the environmental variables (see supporting information on “A priori selection of predictor variables”).

Despite the fact that the existence of significant models with some degree of predictive power suggests that this line of reasoning was successful, it is possible that the predictive power of models would have benefited from access to comprehensive data on relevant predictor variables with a spatial resolution matching that of growth estimates. One such possibility for future modeling could be the use of satellite images for providing a better spatial resolution of temperature, chlorophyll, etc. However, this kind of information was not available and may also be subject to limitations near the coasts as the optical complexity of coastal waters is large compared to the open ocean (Moses et al. 2009). Having said this, the use of satellite data is promising and has been used successfully for modeling bivalve growth in individual‐based models (Thomas et al. 2011; Filgueira et al. 2013).

Scientifically based, spatially explicit information, and user‐friendly maps of important ecological functions are fundamental for any attempts to implement marine spatial planning and therefore to the development of ecosystem‐based management (e.g., Douvere 2008; Guerry et al. 2012; Crossman et al. 2013; but see Hauck et al. 2013 for cautions about over‐reliance on maps). We measured and modeled soft tissue growth of mussels, which directly affects production potential in an aquaculture context and the potential for nutrient removal in the context of mitigating effects of eutrophication. Thus, mussel growth is strongly linked to the potential for the system to perform important provisioning and regulating ecosystem services (Dame 1996; Gallardi 2014; Filgueira et al. 2015) and maps indicating areas of high and low growth potential are therefore of obvious importance for a range of stakeholders engaged in a regional planning process. Note that these estimates could not have been achieved solely by studying patterns of biodiversity, but requires the explicit measurement of processes. As an example of its use, the resulting map of growth produced can be compared to maps of areas identified by Swedish status assessments of ecological status according to the WFD. These comparisons show that areas most in need of mitigation efforts are those where growth is generally high. This indicates that conditions for mussel farming and thus nutrient removal are favorable in these areas (Lindahl et al. 2005; Gren et al. 2009). Furthermore, the map provides planning authorities and farmers with knowledge important for site selection, which is a general issue for planning of aquaculture operations in marine environments (Brigolin et al. 2006; Silva et al. 2011; Filgueira et al. 2014). Results like these can not only be used as practical tools for optimizing rates of production in aquaculture and efficiency of mitigation efforts, but also in a very concrete way expose and resolve conflicts with other user interests, such as tourism, recreation, shipping, and fishing (Douvere and Ehler 2009; Stelzenmüller et al. 2013).

To summarize, the need for spatially explicit mapping of the distribution of marine habitats, species, and ecosystem goods and services is abundantly echoed in contemporary policy developments on regional and national levels of management (e.g., European Commission 2008, 2014). Developing models and integrative frameworks, for synthesizing spatially and temporally fragmented data, are necessary components for the operationalization of these policies (e.g., Guerry et al. 2012; Lindegarth et al. 2014). In this context, the present study illustrates a general methodology for estimating, modeling, and mapping ecological processes influenced by a complex set of biological, chemical, and physical processes.

## Conflict of Interest

None declared.

## Supporting information


**Data S1** A priori selection of predictor variables.
**Figure S1** Distribution of the 13 stations for which both measured and modelled data was available.
**Figure S2** Coefficient of determination (R^2^) for yearly means of observed and modelled data of nine water variables. Error bars show standard deviation among years.
**Figure S3** Coefficient of determination (R^2^) of correlations between monthly means of observed and modelled data for nine water parameters.
**Figure S4** Spearman's rank correlations (*ρ*) between yearly and monthly means of observed data from different years. Error bars represent standard deviation among years.
**Figure S5** Examples of correlations for A) salinity and B) chlorophyll *a* between different years.
**Figure S6** Examples of variation in correlations for 6 different months between years, here 2007 and 2008, for A) temperature, B) salinity, C) chlorophyll *a* and D) total nitrogen.
**Table S1** Spearman's rank correlation coefficients (below diagonal) and correlation plots (above diagonal) among environmental variables used in the final models. Significant correlations in bold.
