# Supplementary material for: Modeling and predicting the growth of the mussel, Mytilus edulis: implications for planning of aquaculture and eutrophication mitigation
Source: Ecol Evol. 2015 Dec 2;5(24):5920–33. doi: 10.1002/ece3.1823 (PMC4717332; doi:10.1002/ece3.1823)
Supplement: Supplementary file 1 — Data S1 A priori selection of predictor variables. Figure S1 Distribution of the 13 stations for which both measured and modelled data was available. Figure S2 Coefficient of determination (R2) for yearly means of observed and modelled data of nine water variables. Error bars show standard deviation among years. Figure S3 Coefficient of determination (R2) of correlations between monthly means of observed and modelled data for nine water parameters. Figure S4 Spearman's rank correlations (ρ) between yearly and monthly means of observed data from different years. Error bars represent standard deviation among years. Figure S5 Examples of correlations for A) salinity and B) chlorophyll a between different years. Figure S6 Examples of variation in correlations for 6 different months between years, here 2007 and 2008, for A) temperature, B) salinity, C) chlorophyll a and D) total nitrogen. Table S1 Spearman's rank correlation coefficients (below diagonal) and correlation plots (above diagonal) among environmental variables used in the final models. Significant correlations in bold. [file ECE3-5-5920-s001.doc]

A PRIORI SELECTION OF PREDICTOR VARIABLES

In order to empirically model and predict spatial patterns of growth in the entire Swedish Skagerrak coast, one fundamental perquisite is to have access to data on predictor variables, not only in sites where growth has been estimated but also from sites where growth must be predicted. The results from a previous study (Bergström et al. 2013) showed that variability in growth of mussels was substantially larger among than within water bodies defined in the Swedish implementation of EU:s “Water Framework Directive”. The study also suggested that spatial patterns among water bodies were qualitatively consistent among years. Based on these arguments, we concluded that predictions about differences in growth among water bodies are justified, and thus that data on predictors with a matching resolution is needed.

While data on potential geographical and geomorphological predictors can be derived from GIS-material, comprehensive data on physico-chemical and biological variables at sufficient resolution are not easily accessible. In order to evaluate the usefulness of modelled data available at the scale of water bodies from the Swedish Meteorological and Hydrological Institute (SMHI; www.vattenwebb.smhi.se), we evaluated (1) the correlation between modelled and observed data of potential predictors at different temporal scales and (2) the temporal consistency of spatial patterns of potential predictors using observed data. The rationale behind these analyses was that to be able to use modelled data on predictors, these should ideally be strongly correlated with observed data and represent variables that show predictable spatial patterns among years. Finally, to improve predictive capacity of future empirical models, (3) collinearity of predictor variables were evaluated and a number of correlated variables were removed from subsequent analyses.

**Evaluating the potential use of modelled data**

We tested the correlation between observed and modelled concentrations of nine environmental variables (temperature, salinity, chlorophyll *a*, nitrate, oxygen, ammonium, phosphate, total nitrogen and total phosphorus) measured in the national monitoring program by the Swedish Meteorological and Hydrological Institute (SMHI). The data come from 13 stations in the area (Fig 1) and modelled data from the matching water body was obtained from www.vattenwebb.smhi.se. The modelled data are an export from a hydrological model for integrated simulations of fluxes and turnover of water and nutrients (‘kustzonsmodellen’). Data from a period of 12 years (2000-2011) was used and the consistency of spatial patterns between measured and modelled concentrations were analysed at the scale of years and months (i.e. years = mean value for each year; months = mean value for each month within each year). With concentrations varying between different water depths, an average concentration was obtained by integrating values within the interval 0 - 20 m taking different volumes (at different depths) into consideration. Only variables that are potentially linked to the growth of bivalves by direct or indirect mechanisms were selected considered.


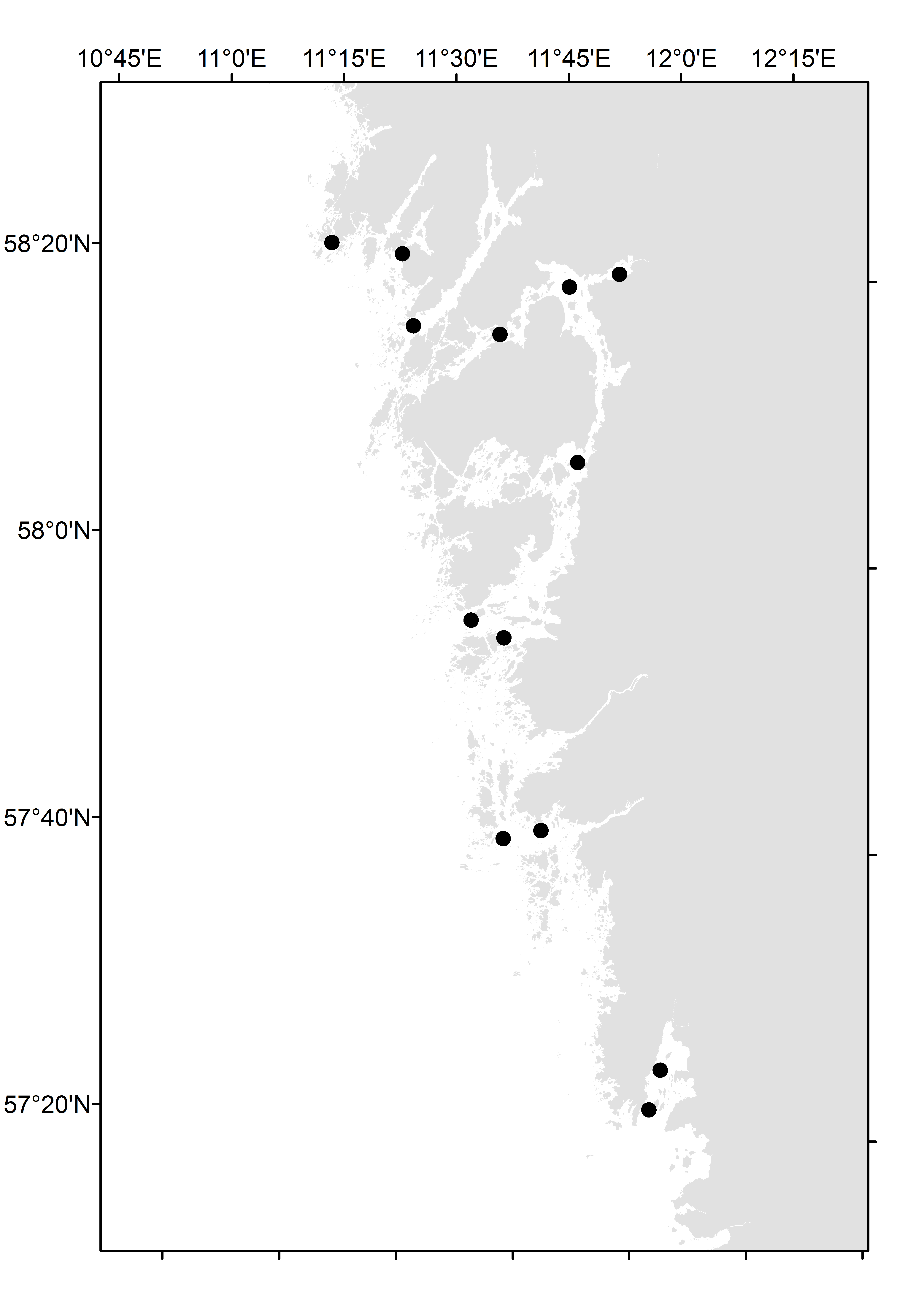


Fig 1. Distribution of the 13 stations for which both measured and modelled data was available.

Correlations between observed and modelled data varied among variables and depending on the scale of aggregation. The strongest correlations were found for spatial patterns of yearly means (Fig 2). Using mean values over all twelve years, aggregated at month (comparing measured and modelled data for each individual month) or year (comparing average measured and modelled data for each year), the strongest link between measured and modelled data were found for salinity (R^2^_year_ = 0.83), total nitrogen (R^2^_year_ = 0.76) and total phosphorus (R^2^_year_ = 0.71) whereas ammonium (R^2^_year_ = 0.10) and temperature (R^2^_year_ = 0.18) had the weakest correlations (Fig 2).


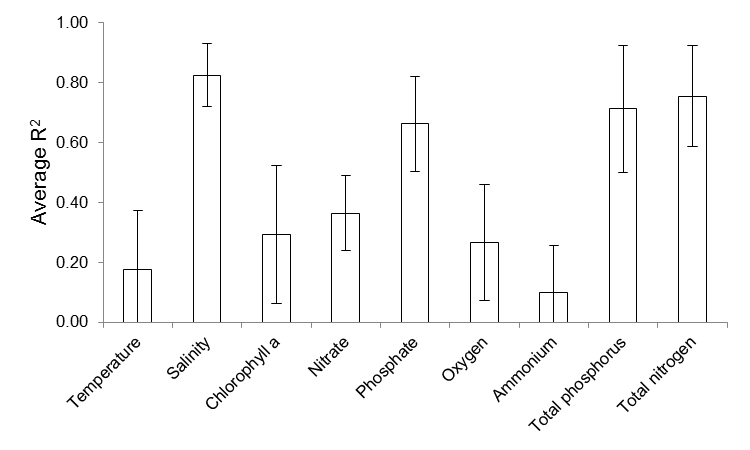


Fig 2. Coefficient of determination (R^2^) for yearly means of observed and modelled data of nine water variables. Error bars show standard deviation among years.

Similar but more complex patterns occur at the scale of months (Fig 3). Overall, the same variables (salinity, total phosphorus and total nitrogen) showed consistent, strong correlations between modelled and observed concentrations at the scale of individual months, particularly when monthly values are averaged over years, giving one mean values per station for the whole period. R^2^ for monthly means of these variables typically vary between 1 and 0.75. For chlorophyll *a*, temperature, nitrate and oxygen, the correlation between modelled and observed values vary strongly among months (Fig 3). In general, it appears that model predictions are more accurate during summer months (May – September) than at other times of the year. Modelled values of ammonium were consistently poorly correlated with measurements.

In conclusion, these first analyses of the potential for using modelled data at a resolution of water bodies as predictors in empirical modelling suggest that salinity, total nitrogen and total phosphorus showed highest consistency between measured and modelled data. These are thus the most promising for use in predictive modelling while other variables such as temperature, chlorophyll *a* and nitrate are less useful for such purpose due to weaker consistency. Nevertheless, significant correlations at certain times of the year suggest that the usefulness of these variables cannot be entirely ruled out.


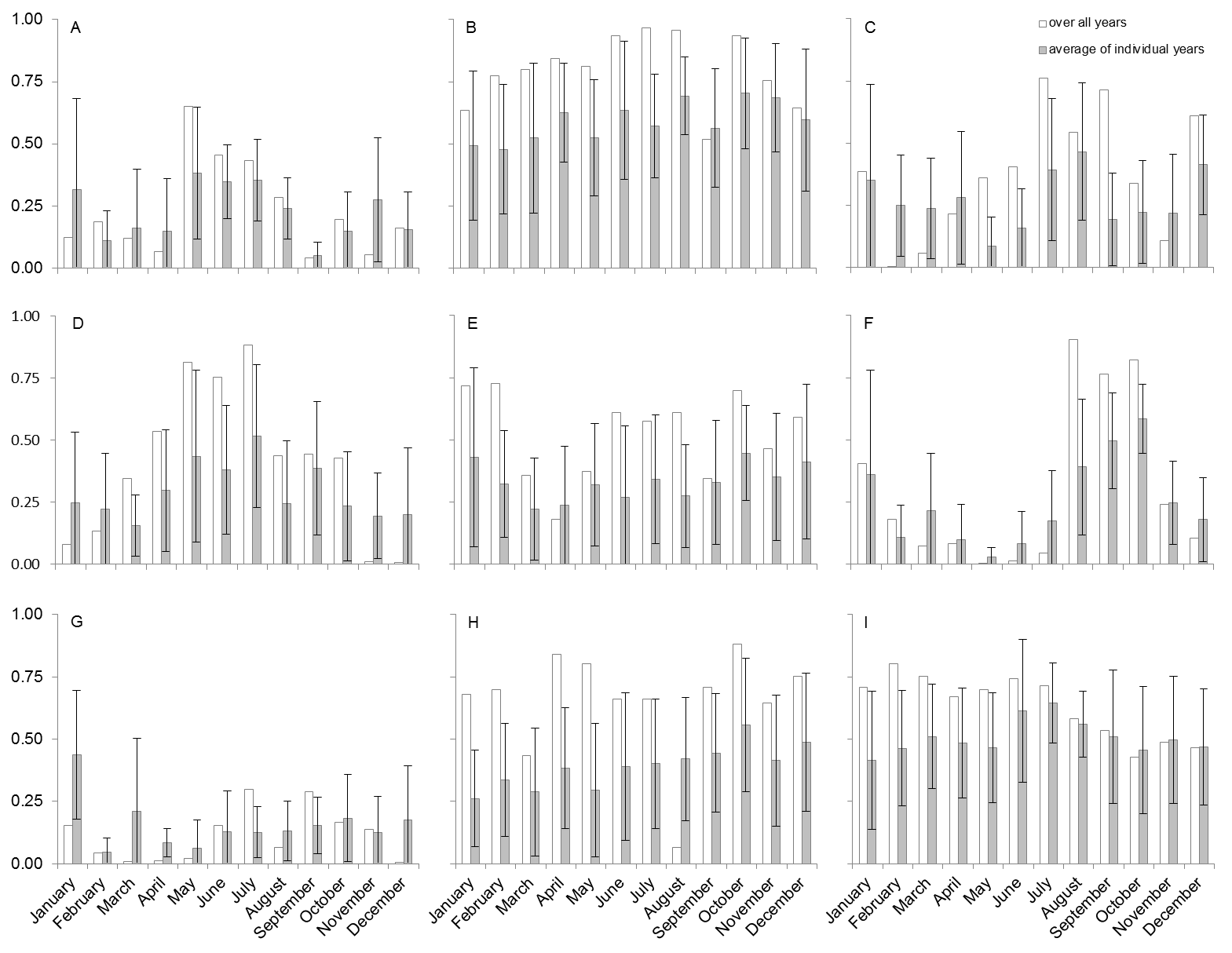


Fig 3. Coefficient of determination (R^2^) of correlations between monthly means of observed and modelled data for nine water parameters. A) temperature, B) salinity, C) chlorophyll *a*, D) nitrate, E) phosphate, F) oxygen, G) ammonium, H) total phosphorus and I) total nitrogen. Shaded bars represent the average of correlations for months in individual years and open bars the correlation between observed and modelled data for individual months averaged over all years. Error bars are standard deviation among years for a particular month.

**Consistency of spatial patterns**

To evaluate temporal consistency of spatial patterns of physico-chemical predictor variables, i.e. the extent to which spatial patterns are stable, we used data on fourteen available water variables collected by SMHI at 21 sites during 12 years (2000-2011) on the Swedish west coast. Data were available from several different depths and for each site and parameter an average concentration for the depth interval 0-20 m was calculated using available information on concentrations and volume estimates. Analysis of temporal consistency of spatial patterns was performed at the scale of months and years, using the average concentrations and Spearman’s rank correlations.


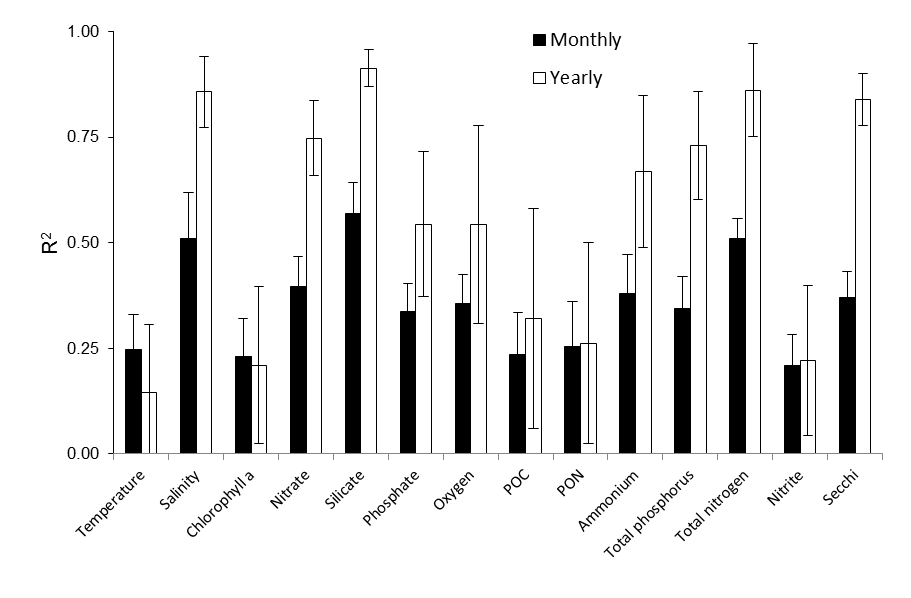


Fig 4. Spearman’s rank correlations (*ρ*) between yearly and monthly means of observed data from different years. Error bars represent standard deviation among years.

Analysis of consistency in spatial patterns showed that several water variables show spatial patterns of concentrations which are consistent over time (Fig 4). In general, the correlation among years was more pronounced at the scale of years than for months. Some exceptions (i.e. temperature, chlorophyll *a* and nitrite) showed average correlations which were equal or slightly larger.

The strongest correlations, at the scale of years, were observed for silicate (*ρ* = 0.91), salinity and total nitrogen (both *ρ* = 0.86) indicating a strong temporal consistency in spatial concentration patterns. Spatial patterns of other variables (e.g. chlorophyll *a* and nitrite) were not temporally consistent with low *ρ* values.

More detailed analyses of the consistency of patterns among years showed that spatial patterns of some variables (e.g. salinity) where not only strongly correlated but also quantitatively consistent among years. Others, with weak correlations were also quantitatively more variable (e.g. chlorophyll *a*) (Fig 5). Similar patterns are observed at the scale of months (Fig 6), where salinity and total nitrogen display a homogenous distribution among different months, while for the less temporally consistent parameters (e.g. temperature and chlorophyll *a*) the picture is quite different.


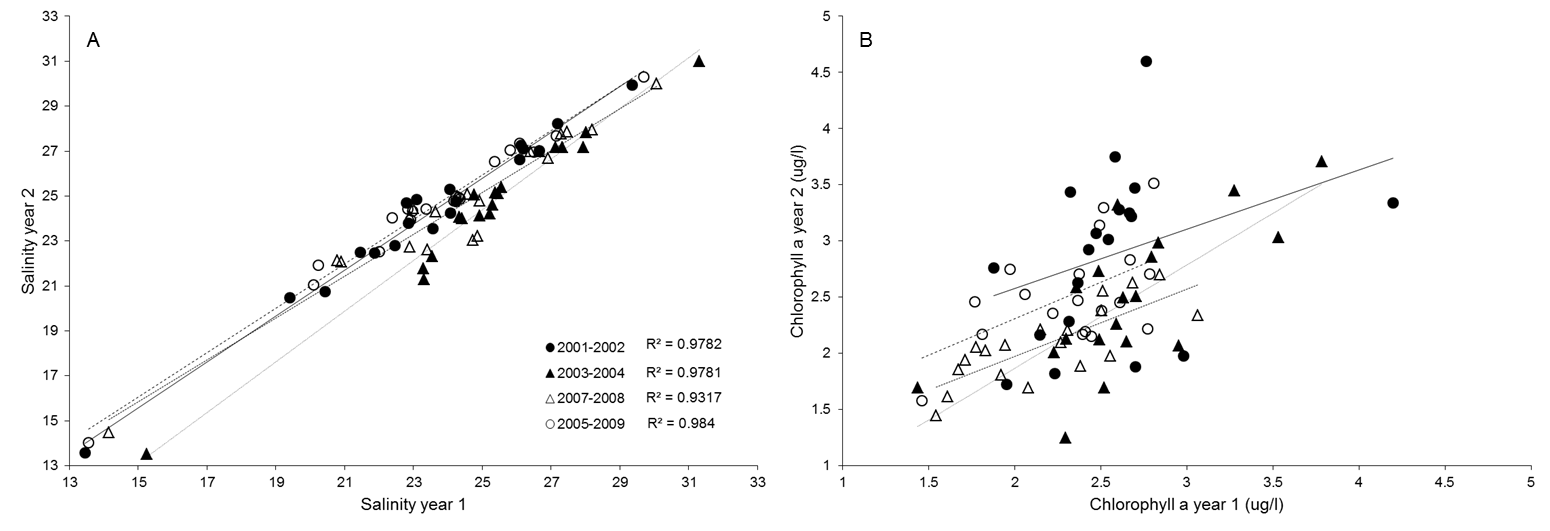


Fig 5. Examples of correlations for A) salinity and B) chlorophyll *a* between different years.


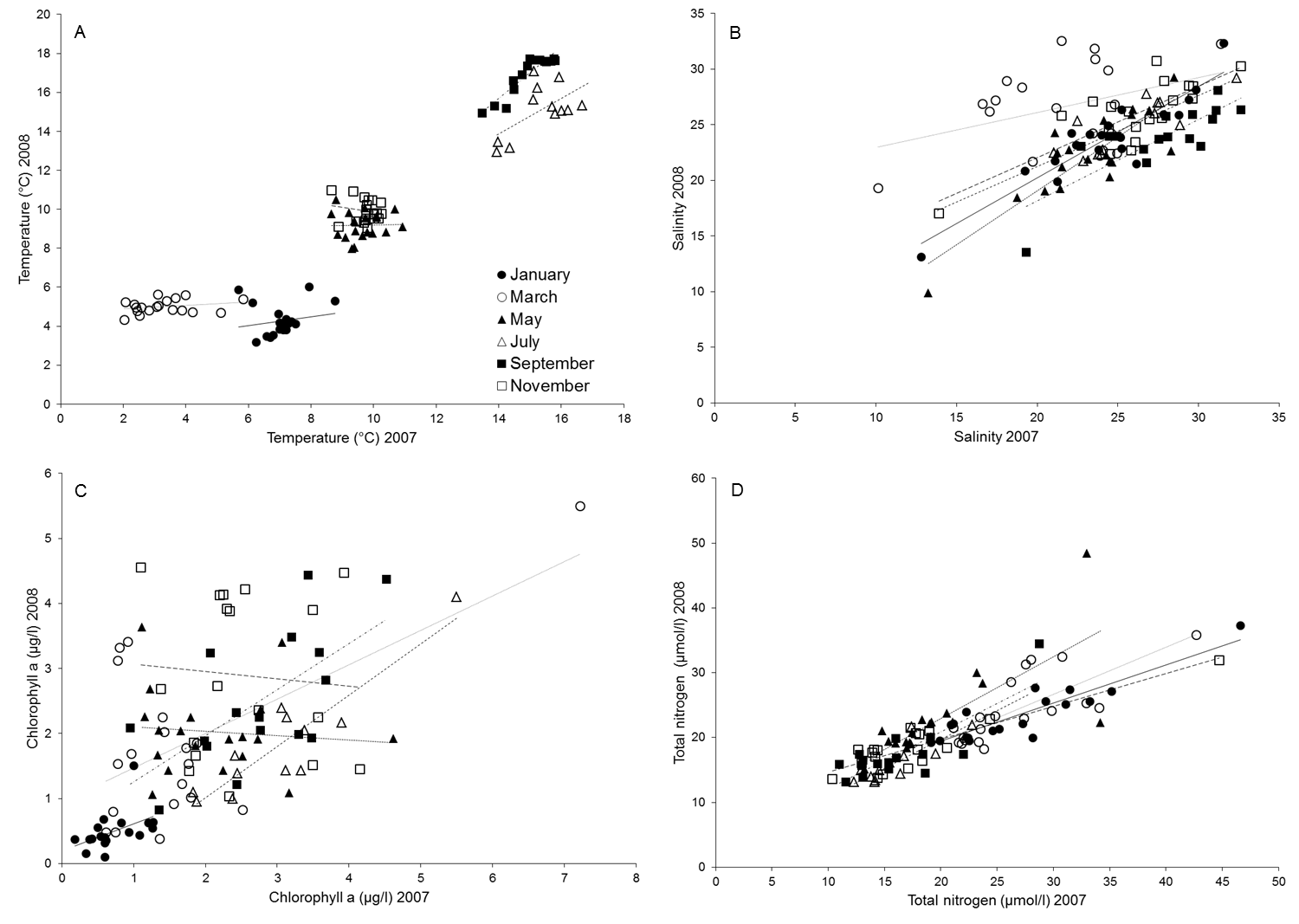


Fig 6. Examples of variation in correlations for 6 different months between years, here 2007 and 2008, for A) temperature, B) salinity, C) chlorophyll *a* and D) total nitrogen.

Thus based on the consistency of spatial patterns, we found strong differences among the different physico-chemical variables. The variables showing the strongest correlations and were salinity, total nitrogen and silicate while ammonium, nitrate total phosphorus, phosphate and oxygen also showed some consistency. Other variables were less consistent and therefore probably less likely to predict temporally consistent patterns. Using yearly averages of concentration were in general more suitable for the use in predictive modelling as the correlations at this scale was higher for almost all parameters. The variables that showed the strongest consistency at the scale of years were also the ones that had the strongest correlations at the scale of months and most of the variables showed similar correlations throughout the different months with a slight depression during spring and autumn.

In conclusion, thee two types of analyses indicated that the variables showing the strongest correlation between measured and modelled data also showed the highest consistency in spatial patterns. Unfortunately chlorophyll *a*, which can be considered the best proxy for the available food for filter-feeders in marine environments, showed weak correlations between observed and modelled data. Similarly, spatial patterns of chlorophyll *a* were not strongly consistent among years. Nevertheless, because both sets of analyses showed significant correlations and due to its fundamental role as a proxy of food availability chlorophyll *a* was included in subsequent empirical modelling.

**Evaluating collinearity among predictors**

Collinearity (e.g. non independence of predictor variables) can cause problems with parameter estimations as a result of inflated variance of regression parameters. Potential collinearity among explanatory variables was investigated using the threshold-based pre-selection technique; Variance Inflation Factor (VIF). Normally a VIF-value less than 10 is not considered a major problem (Myers 1990; Belsey 1991; Hair et al. 1995) while a common rule of thumb is that a value higher than 5 warrant further investigation (Rogerson 2001). Before analysing the VIF we grouped the available explanatory variables into five different environmental categories (geographical, oceanographical, hydrological, chemical and biological) that reflected their different function. We then selected a value of 3 (within variable category) and 6 (between variable categories) as limit for potential inclusion in modelling procedure and explanatory variables with higher values were removed prior to modelling to reduce the problem of collinearity. However, variables believed to represent different distinct ecological processes were included even if the variance inflation factor was above 3 since if two variables have a suppressor relationship, removing one will result in an under-/overestimation of the effect of the remaining predictor. Variation inflation factor analysis reduced the number of potential explanatory variables from 15 to 11 by removing the variables oxygen, nitrate, phosphate and surface area.

Finally, after analysing the possibility to utilise modelled data as explanatory variables, evaluating the consistency of spatial patterns, and investigating the collinearity among variables a total of 11 variables potential for the use in predictive modelling remained. These variables were further investigated and the correlations among the retained variables were generally low (Table 1). Although some strong (and several weak) and significant correlations persisted. The strongest positive correlations found were between chlorophyll *a* and total amount of nitrogen (*ρ* = 0.77) closely followed by latitude-salinity (*ρ* = 0.75) whereas total nitrogen-salinity (*ρ *= -0.66) and temperature-ammonium (*ρ* = -0.65) displayed the main negative correlations in the final set of explanatory variables. Interestingly no significant correlations could be found between chlorophyll *a* and either temperature, total amount of phosphorus or ammonium (Table 1).

**Table 1. Spearman’s rank correlation coefficients (below diagonal) and correlation plots (above diagonal) among environmental variables used in the final models. Significant correlations in bold.**

|  | **Geographical** | | **Oceanographical** | | | **Hydrological** | | | **Chemical** | | | | **Biological** |
| --- | --- | --- | --- | --- | --- | --- | --- | --- | --- | --- | --- | --- | --- |
| **Variable** | *Latitude* | *Distance* | *Volume* | *Turnover* | *Exposure* | | *Salinity* | *Temperature* | *Total Nitrogen* | *Total Phosphorous* | *Ammonium* | *Chlorophyll a* | |
| *Latitude* | 1 | 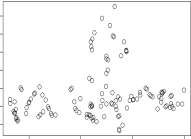 | 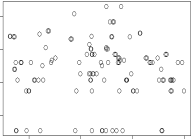 | 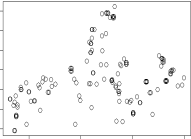 | 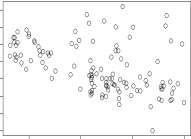 | | 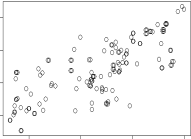 | 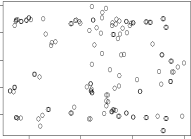 | 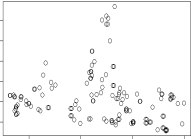 | 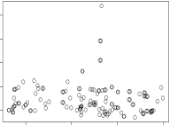 | 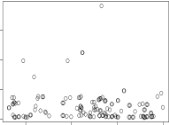 | 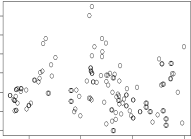 | |
| *Distance* | 0.25 | 1 | 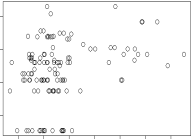 | 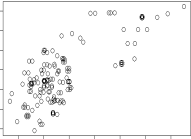 | 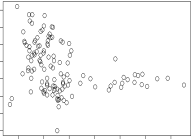 | | 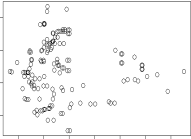 | 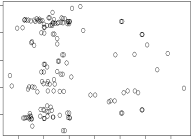 | 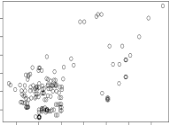 | 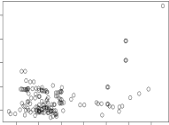 | 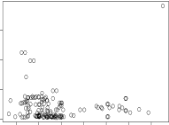 | 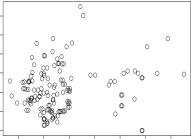 | |
| *Volume* | -0.05 | 0.29 | 1 | 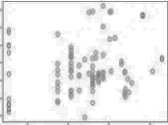 | 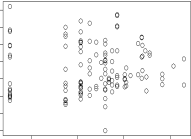 | | 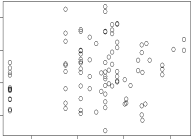 | 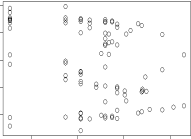 | 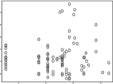 | 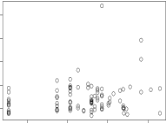 | 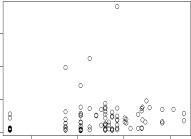 | 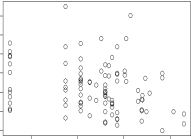 | |
| *Turnover* | 0.26 | 0.59 | 0.21 | 1 | 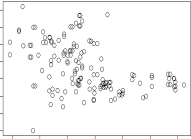 | | 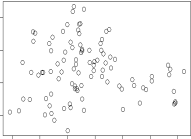 | 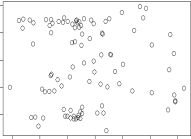 | 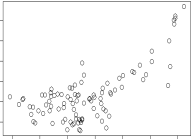 | 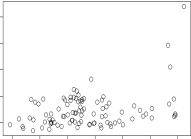 | 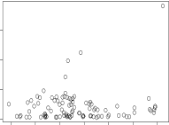 | 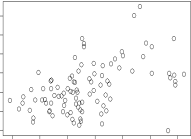 | |
| *Exposure* | -0.44 | -0.40 | -0.14 | -0.44 | 1 | | 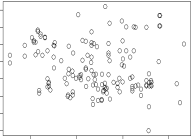 | 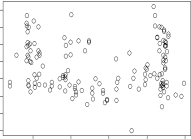 | 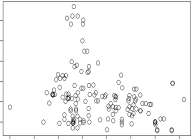 | 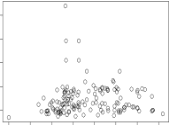 | 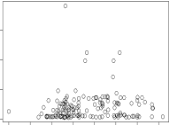 | 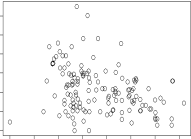 | |
| *Salinity* | 0.75 | 0.06 | 0.12 | 0.01 | -0.17 | | 1 | 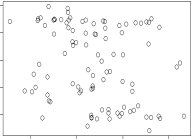 | 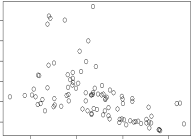 | 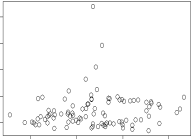 | 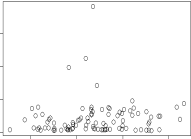 | 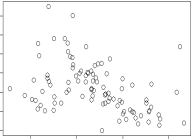 | |
| *Temperature* | -0.06 | 0.01 | -0.27 | -0.04 | -0.07 | | 0.28 | 1 | 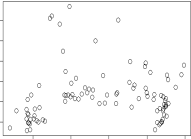 | 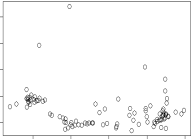 | 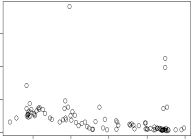 | 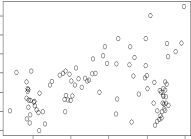 | |
| *Total Nitrogen* | -0.26 | 0.28 | 0.02 | 0.49 | -0.66 | | 0.15 | -0.28 | 1 | 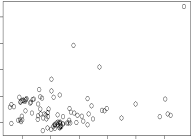 | 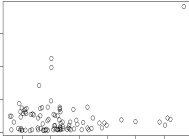 | 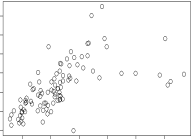 | |
| *Total Phosphorus* | -0.18 | 0.06 | 0.15 | 0.16 | 0.15 | | -0.25 | 0.17 | -0.22 | 1 | 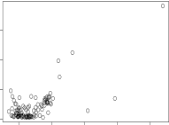 | 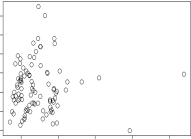 | |
| *Ammonium* | -0.17 | -0.17 | 0.34 | -0.01 | 0.08 | | -0.65 | 0.20 | -0.02 | 0.44 | 1 | 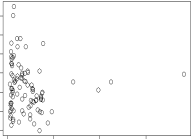 | |
| *Chlorophyll a* | -0.16 | 0.14 | -0.25 | 0.48 | 0.11 | | -0.32 | 0.77 | -0.05 | -0.04 | -0.07 | 1 | |


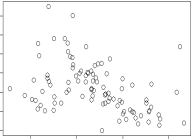
REFERENCES

1

Belsey, D. A. (1991) Conditioning diagnostics: collinearity and weak data regression. Wiley.

Bergström, P., Lindegarth, S. & Lindegarth, M. (2013) Temporal consistency of spatial pattern in growth of the mussel, *Mytilus edulis*: implications for predictive modelling. Estuarine, Coastal and Shelf Science*,* **131**, 93-102

Hair, J. F., Anderson, R. E., Tatham, R. L. & Black, W. C. (1995) Multivariate Data Analysis. 3 edition. Macmillan, New York.

Myers, R. H. (1990) Classical and modern regression with applications. PWS-KEN Publishing Company, Boston.

Rogerson, P. A. (2001) Statistical methods for geography. Sage, London.
